# Adherence to the dietary approaches to stop hypertension diet and all-cause mortality in patients with a history of heart failure

**DOI:** 10.3389/fnut.2022.1015290

**Published:** 2022-09-27

**Authors:** Ting-Yu Chou, Wei-Ju Liu, Chia-Lin Lee, Jun-Sing Wang

**Affiliations:** ^1^Department of Education, Taichung Veterans General Hospital, Taichung, Taiwan; ^2^Department of Medical Research, Taichung Veterans General Hospital, Taichung, Taiwan; ^3^Division of Endocrinology and Metabolism, Department of Internal Medicine, Taichung Veterans General Hospital, Taichung, Taiwan; ^4^Department of Medicine, School of Medicine, National Yang Ming Chiao Tung University, Taipei, Taiwan; ^5^Department of Post-baccalaureate Medicine, College of Medicine, National Chung Hsing University, Taichung, Taiwan; ^6^Rong Hsing Research Center for Translational Medicine, Institute of Biomedical Science, National Chung Hsing University, Taichung, Taiwan

**Keywords:** DASH, heart failure, mortality, NHANES, sodium

## Abstract

**Background and aims:**

We investigated the association of adherence to the Dietary Approaches to Stop Hypertension (DASH) diet with all-cause mortality in patients with a history of heart failure.

**Methods:**

We analyzed data from the National Health and Nutrition Examination Survey (NHANES). Dietary information was obtained from a 24-h dietary recall interview. Adherence to the DASH diet was assessed using the DASH score. The primary outcome was all-cause mortality which was confirmed by the end of 2011. Weighted Cox proportional hazards regression models were used to determine the hazard ratios and 95% CI for the association of the DASH score and all-cause mortality with multivariate adjustment.

**Results:**

The median DASH score was 2 among the 832 study participants. There were 319 participants who died after a median follow-up duration of 4.7 years. A higher DASH score (>2 vs. ≤ 2) was not associated with a decrease in the risk of all-cause mortality (adjusted HR 1.003, 95% CI 0.760–1.323, *p* = 0.983). With respect to the components of the DASH score, a lower sodium intake was not associated with a decreased risk of mortality (adjusted HR 1.045, 95% CI 0.738–1.478, *p* = 0.803).

**Conclusion:**

A higher DASH score (>2 vs. ≤ 2) was not associated with all-cause mortality in patients with heart failure.

## Introduction

The majority of deaths worldwide were attributed to non-communicable diseases (1). A healthy lifestyle, including dietary modifications, may help control risk factors and healthcare burdens of non-communicable diseases (2, 3). For instance, dietary salt reduction may lower blood pressure (4) and reduce long-term mortality risk non-communicable diseases (5). The Dietary Approaches to Stop Hypertension (DASH) diet has been recommended as a dietary modification that has effects on both blood pressure and blood cholesterol (6). The DASH diet is rich in fruits, vegetables, and low-fat dairy foods. These foods are high in potassium, magnesium, calcium, and fiber (7, 8). Combining the DASH dietary pattern with sodium reduction could reduce blood pressure and non-communicable diseases (6–9).

Among non-communicable diseases, the burden of heart failure is increasing (10, 11) and the quality of medical care for affected patients remains suboptimal (12). Guidelines recommend adopting a healthy diet as part of the management for heart failure; however, recommendations for specific dietary patterns are lacking (13). Dietary sodium restriction is frequently recommended to patients with heart failure (14) and is endorsed by most treatment guidelines (15, 16). Although the DASH diet has been associated with a reduced risk of mortality in the general population (17–19) and in patients with hypertension (20), its effect on patients' outcomes in heart failure is not yet clear. In this study, we examined the association between a DASH dietary pattern and all-cause mortality in patients with a history of heart failure using data from the National Health and Nutrition Examination Survey (NHANES).

## Materials and methods

**Figure 1:**
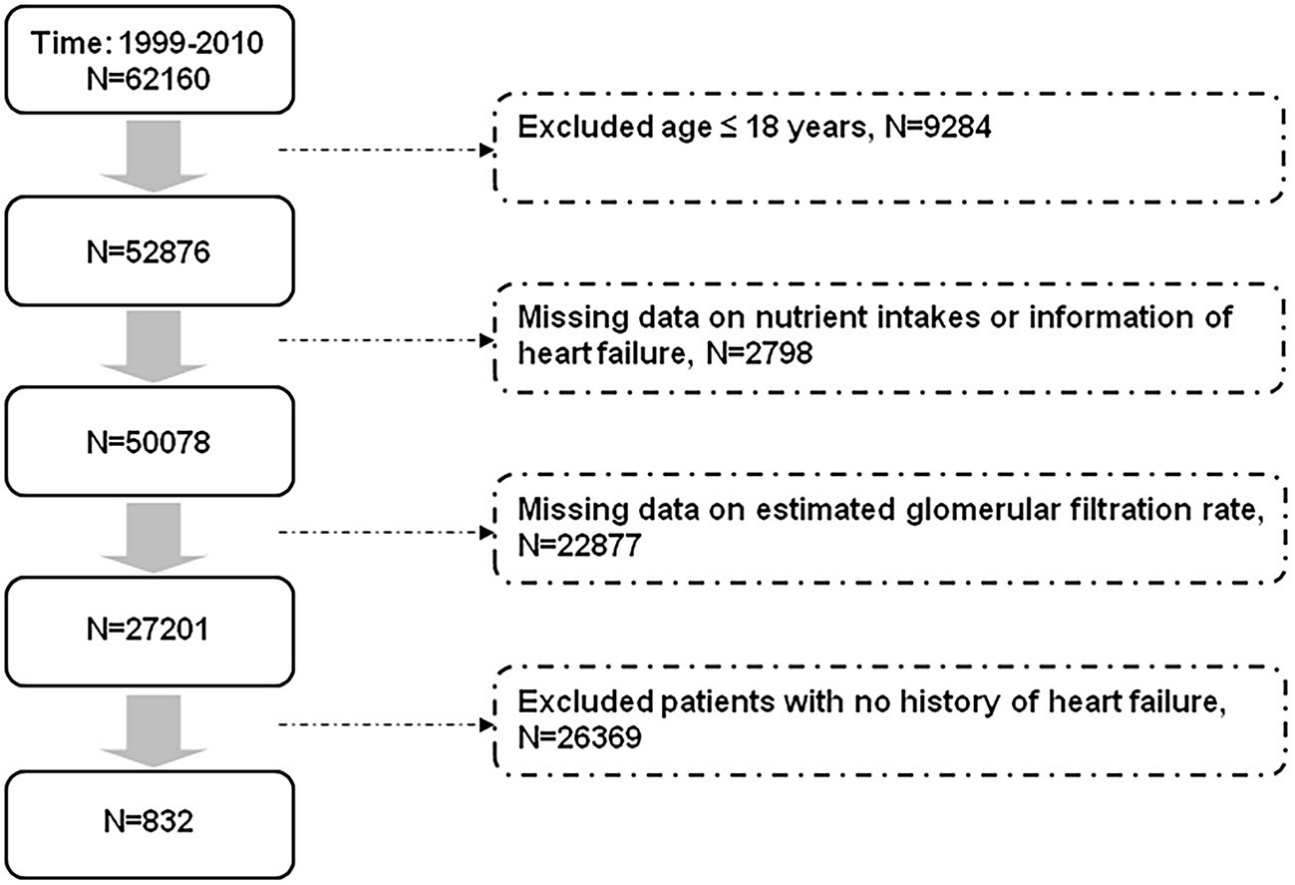
Flow diagram displaying the selection of study population.

We selected our study population from the participants in the NHANES, which was conducted to assess the nutritional and health status of the US population. Briefly, data were collected through personal interviews, physical examinations, laboratory tests, and questionnaires (https://www.cdc.gov/nchs/surveys.htm). The selection of patients with heart failure is shown in Figure 1. We excluded participants aged ≤ 18 years, with missing information on nutrient intakes and renal function, with unknown history about heart failure, and those who reported no history of heart failure from the 62,160 participants [mean age 36.1 (95% CI 35.6–36.5) years] in the NHANES from 1999 to 2010. Finally, we included 832 participants with a history of heart failure. Survival status of the study population by the end of 2011 was confirmed according to information from the National Death Index.

The conduction of this study followed the Declaration of Helsinki. Our protocol was approved by the Institutional Review Board of Taichung Veterans General Hospital, Taichung, Taiwan (approval number: CE18312A). Informed consent was provided by all of the NHANES participants. Dietary information of the NHANES participants were obtained from a 24-h dietary recall interview, which was conducted in person by trained interviewers (https://www.cdc.gov/nchs/nhanes/measuring_guides_dri/measuringguides.htm). A computer-assisted software program, and measurement aids and visuals including charts and drawings were used to facilitate the 24-h dietary recall interview. Data from the 24-h dietary recall interview were translated for analyses according to the Food Patterns Equivalents Database (developed by the United States Department of Agriculture (https://www.ars.usda.gov/northeast-area/beltsville-md-bhnrc/beltsville-human-nutrition-research-center/food-surveys-research-group/docs/fped-databases/), which had been applied to the NHANES population (21–23).

We used the DASH score (20) to assess the dietary concordance to the DASH diet. To determine the DASH score, data on 9 target nutrients (Table 1) (20) were obtained based on information from the 24-h dietary recall interview. We determined whether the participants' intake met the goal of each target nutrient. This method has been applied to the NHANES population (21–23). One point was assigned to each target nutrient if the study participants' intake met the goal (20, 23, 24), while half (0.5) a point was assigned if only the intermediate goal was met. The maximum DASH score is 9, and a higher score indicates better concordance with the DASH diet. We determined the study participants' renal function (estimated glomerular filtration rate, eGFR) using a well-documented method (25).

**Table 1:** Nutrients target for DASH score and nutrients intake of the study population.

**Nutrients**	**DASH target (score = 1)**	**Intermediate target (score = 0.5)**	**Study population [mean (95% CI)]**
Saturated fat (% of energy)	< 6	< 11	11.1 (10.8–11.4)
Total fat (% of energy)	< 27	< 32	33.8 (33.0–34.5)
Protein (% of energy)	>18	>16.5	15.9 (15.4–16.4)
Cholesterol (mg/1,000 kcal)	< 71.4	< 107.1	262.8 (244.7–280.9)
Fiber (g/1,000 kcal)	>14.8	>9.5	13.9 (13.2–14.7)
Magnesium (mg/1,000 kcal)	>238	>158	246.3 (235.6–257.0)
Calcium (mg/1,000 kcal)	>590	>402	762.3 (717.9–806.6)
Potassium (mg/1,000 kcal)	>2,238	>1,534	2,426 (2,321–2531)
Sodium (mg/1,000 kcal)	< 1,143	< 1,286	2,903 (2,757–3,049)

DASH, Dietary Approaches to Stop Hypertension.

We used the Statistical Analysis System survey procedures (SAS version 9.4, 2013, Cary, NC, USA) to conduct all of the statistical analyses, which were adequately weighted (https://wwwn.cdc.gov/nchs/nhanes/analyticguidelines.aspx). We divided the study population into two groups according to their DASH score (>median vs. ≤ median). Between-group differences in categorical and continuous variables were examined using the Chi-square test and independent *t* test, respectively. The primary outcome was all-cause mortality. We conducted weighted Cox proportional hazards regression models to determine the hazard ratios (HR) and 95% CI for the association of DASH score (>median vs. ≤ median) and all-cause mortality, with multivariate adjustment. Adjusted variables included known factors related to risk of mortality (age, sex, race, body mass index, smoking, systolic blood pressure, diabetes, and eGFR) and dietary factor (daily energy intake). The association was also examined in subgroups of body mass index (≥30 vs. < 30 kg/m^2^), daily energy intake (≥25 vs. < 25 kcal/kg/day), glucose regulation status (diabetes vs. no diabetes), and renal function (eGFR ≥ 60 vs. < 60 ml/min/1.73 m^2^). We conducted Cubic spline of DASH score vs. all-cause mortality risk as a sensitivity test. Finally, the associations of the 9 parts of the DASH score (20) with all-cause mortality were determined. A two-sided *P-*value < 0.05 was considered statistically significant in all of the statistical analyses.

## Results

Table 1 shows the nutrient targets for the 9 parts of the DASH score, and the nutrient intakes of the study population. Overall, the study population had suboptimal concordance with the DASH diet (median DASH score = 2). Participants with a higher DASH score (>2 vs. ≤ 2, Table 2) had a lower body mass index, a higher systolic blood pressure, and a lower total cholesterol and triglyceride with a higher high-density lipoprotein cholesterol, compared with those who had a lower DASH score. The former group also had a lower intake of daily calories, which comprised a lower proportion from fat and higher proportions from carbohydrate and protein, compared with the latter group. There were only modest between-group differences for the other variables.

**Table 2:** Characteristics of study participants according to DASH scores.

	**DASH score**		
**Variables**	** ≤ 2**	**>2**	***P*-value**
Number of participants	466	366	
Age, years	65.7 (63.8–67.6)	65.5 (64.0–67.0)	< 0.001
Male, *n* (%)	265 (53.5)	214 (56.0)	0.551
Body mass index, kg/m^2^	31.5 (30.6–32.4)	30.3 (29.4–31.2)	< 0.001
Systolic blood pressure, mm Hg	129.4 (126.8–132.0)	133.2 (130.5–136.0)	< 0.001
Diastolic blood pressure, mm Hg	66.6 (64.2–69.0)	68.1 (66.1–70.0)	< 0.001
Hypertension, *n* (%)	340 (70.8)	273 (67.5)	0.414
Smoking, *n* (%)	299 (63.7)	225 (61.5)	0.534
Total cholesterol, mmol/L	4.95 (4.80–5.09)	4.82 (4.66–4.97)	< 0.001
HDL cholesterol, mmol/L	1.26 (1.22–1.31)	1.27 (1.21–1.34)	< 0.001
Triglycerides, mmol/L	1.94 (1.81–2.08)	1.89 (1.72–2.07)	< 0.001
Fasting plasma glucose, mmol/L	6.38 (6.13–6.64)	6.58 (6.15–7.00)	< 0.001
HbA1c, %	6.07 (5.94–6.19)	6.13 (5.98–6.28)	< 0.001
eGFR, mL/min/1.73 m^2^	70.5 (66.9–74.1)	70.5 (66.6–74.3)	< 0.001
**Daily calories, kcal/day**	1,828 (1,751–1,906)	1,696 (1,565–1,827)	< 0.001
% from carbohydrate	47.2 (46.3–48.2)	54.5 (53.2–55.8)	
% from fat	37.9 (37.0–38.7)	28.4 (27.5–29.2)	
% from protein	14.9 (14.5–15.4)	17.2 (16.4–17.9)	

Data are presented as mean (95% CI) or n (%). DASH, Dietary Approaches to Stop Hypertension. eGFR, estimated glomerular filtration rate. HbA1c, glycated hemoglobin. HDL, high-density lipoprotein.

**Figure 2:**
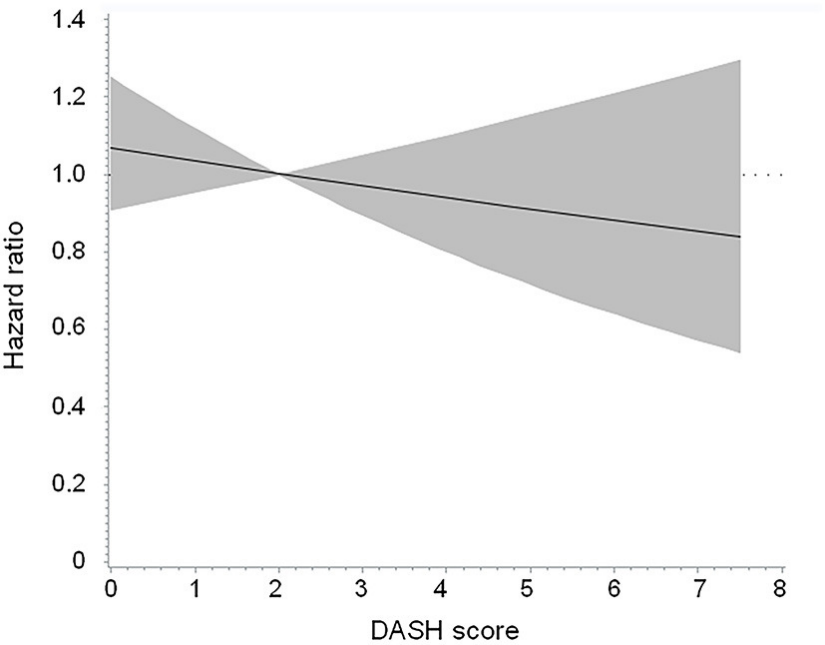
Cubic spline of DASH score vs. risk of all-cause mortality in the overall population. The *p*-value was 0.423 for testing for linear relation. DASH, Dietary Approaches to Stop Hypertension.

There were 319 participants who died after a median follow-up duration of 4.7 years (mortality rate 69.5 per 1,000 person-years). Table 3 shows a null association between a higher DASH score (>2 vs. ≤ 2) and all-cause mortality (adjusted HR 1.003, 95% CI 0.760 to 1.323, *p* = 0.983). The findings were similar in the subgroups of age (≥65 vs. < 65 years), sex (male vs. female), body mass index (≥30 vs. < 30 kg/m^2^), daily energy intake (≥25 vs. < 25 kcal/kg/day), glucose regulation status (diabetes vs. no diabetes), and renal function (eGFR ≥ 60 vs. < 60 ml/min/1.73 m^2^). Figure 2 shows the Cubic spline of DASH score vs. risk of all-cause mortality. There was a modest decrease (but not significant, *p* = 0.423) in all-cause mortality risk with an increase in DASH score.

**Table 3:** Association of DASH score (>2 vs. ≤ 2) with all-cause mortality in various subgroups.

	**DASH score ≤ 2**	**DASH score > 2**	***P*-value**
Overall (*n =* 832)	Reference	HR 1.003 (0.760, 1.323)^a^	0.983
Age < 65 years (*n =* 283)	Reference	HR 0.617 (0.269, 1.415)^a^	0.251
Age ≥ 65 years (*n =* 549)	Reference	HR 1.039 (0.762, 1.417)^a^	0.807
Male (*n =* 479)	Reference	HR 0.969 (0.641, 1.465)^a^	0.880
Female (*n =* 353)	Reference	HR 1.059 (0.740, 1.515)^a^	0.753
Body mass index < 30 kg/m^2^ (*n =* 446)	Reference	HR 1.041 (0.767, 1.414)^a^	0.793
Body mass index ≥ 30 kg/m^2^ (*n =* 374)	Reference	HR 0.861 (0.526, 1.409)^a^	0.548
Calorie intake < 25 kcal/kg/day (*n =* 594)	Reference	HR 0.959 (0.699, 1.314)^a^	0.791
Calorie intake ≥ 25 kcal/kg/day (*n =* 238)	Reference	HR 0.931 (0.526, 1.647)^a^	0.804
No diabetes (*n =* 466)	Reference	HR 1.088 (0.764, 1.550)^a^	0.636
Diabetes (*n =* 366)	Reference	HR 0.852 (0.588, 1.234)^a^	0.393
eGFR ≥ 60 mL/min/1.73 m^2^ (*n =* 502)	Reference	HR 1.032 (0.710, 1.501)^a^	0.867
eGFR < 60 mL/min/1.73 m^2^ (*n =* 330)	Reference	HR 0.902 (0.599, 1.358)^a^	0.618

DASH, Dietary Approaches to Stop Hypertension. eGFR, estimated glomerular filtration rate. ^*a*^Hazard Ratio (95% CI), adjusted for age, sex, race, body mass index, smoking, systolic blood pressure, diabetes, estimated glomerular filtration rate, and daily energy intake.

Table 4 shows the associations of the 9 target nutrients with all-cause mortality. We observed that concordance with each component of the DASH score was not associated with a significant difference in all-cause mortality risk (all *p* > 0.1). Notably, a lower sodium intake (< 1,143 mg/1,000 kcal) was not associated with a decrease in mortality risk in the study population (adjusted HR 1.045, 95% CI 0.738 to 1.478, *p* = 0.803).

**Table 4:** Associations of DASH score components with all-cause mortality.

	**Score = 0**	**Score = 1**	***P*-value**
Saturated fat	Reference	HR 1.028 (95% CI 0.722, 1.463)^a^	0.876
Total fat	Reference	HR 1.010 (95% CI 0.748, 1.363)^a^	0.948
Protein	Reference	HR 0.993 (95% CI 0.736, 1.339)^a^	0.962
Cholesterol	Reference	HR 1.136 (95% CI 0.823, 1.569)^a^	0.433
Fiber	Reference	HR 0.705 (95% CI 0.427, 1.166)^a^	0.171
Magnesium	Reference	HR 0.624 (95% CI 0.346, 1.127)^a^	0.116
Calcium	Reference	HR 0.900 (95% CI 0.647, 1.250)^a^	0.524
Potassium	Reference	HR 0.698 (95% CI 0.426, 1.143)^a^	0.151
Sodium	Reference	HR 1.045 (95% CI 0.738, 1.478)^a^	0.803

DASH, Dietary Approaches to Stop Hypertension. ^*a*^adjusted for age, sex, body mass index, and daily energy intake.

## Discussion

We demonstrated that a higher DASH score (>2 vs. ≤ 2) was not associated with risk of all-cause mortality (adjusted HR 1.003, 95% CI 0.760 to 1.323, *p* = 0.983) in patients with a history of heart failure during a median follow-up duration of 4.7 years. The findings were consistent across various subgroups (age, sex, body mass index, calorie intake, diabetes, and chronic kidney disease). Our results suggest that more evidence is needed for recommendation of the DASH diet for patients with heart failure to improve their outcomes.

Adopting a DASH dietary pattern has been associated with a lower all-cause mortality rate in the general population (17–19) and in patients with hypertension (20). Assessment of sodium intake is one of the components of the DASH score (20). Furthermore, dietary sodium restriction to prevent fluid overload has been endorsed as the cornerstone of therapy for patients with heart failure since the early 1950s (26). Nevertheless, it is not yet clear whether a dietary pattern concordance with the DASH diet helps reduce the mortality rate in patients with heart failure. In a cohort study of women who had experienced a hospitalization for heart failure (27), a higher DASH score was associated with a modest reduction in all-cause mortality (adjusted HR 0.84, 95% CI 0.70 to 1.00, *p* = 0.010). However, findings regarding the association between concordance with the DASH diet and incident heart failure were not consistent (28–33). We did not observe an association between the DASH score and all-cause mortality risk in patients with a history of heart failure (Table 3; Figure 2). Even if we selected participants who had a DASH score >4.5 and compared them with those who had a DASH score ≤ 2, there was no significant between-group difference in the risk of all-cause mortality (HR 0.838, 95% CI 0.383, 1.835, *p* = 0.656). In this context, the current evidence is insufficient to recommend the DASH diet for patients with heart failure to improve their outcomes.

We further investigated the associations of the DASH score components with all-cause mortality in our study population. It is worth noting that sodium intake < 1,143 mg/1,000 kcal (score = 1) was not associated with a lower risk of all-cause mortality (Table 4). In a previous study which revealed a risk reduction for incident heart failure with an increase in DASH score (29), the mean sodium intakes were similar (~2,500 mg/day) across the DASH score quartiles. Whether a restriction in dietary sodium reduces the risk of cardiovascular diseases and mortality remains a subject of debate (34). A urinary sodium excretion rate of < 3.0 g/day (vs. 4.0–6.0 g/day) was associated with an increased risk of cardiovascular diseases or death in a large cohort study (35). Similar findings were noted regarding the associations of urinary sodium excretion and dietary sodium restriction with adverse heart failure outcomes (36–38). Our findings were in line with these reports, and were further supported by a recent randomized controlled trial (39) in which a low sodium diet (< 1,500 mg/day vs. usual care) was not associated with a risk reduction in clinical outcomes in patients with chronic heart failure. These results may be partly explained by some detrimental effects associated with low sodium intake, such as decreased cardiac output and renal perfusion, and activation of the renin–angiotensin–aldosterone system (40). Hence, whether or not to recommend a low sodium diet for patients with heart failure deserves careful consideration.

In contrast, an increase in urinary potassium excretion attenuated the cardiovascular risk associated with high urinary sodium excretion (41). We observed a modest, but non-significant, reduction in all-cause mortality risk (adjusted HR 0.698, 95% CI 0.426 to 1.143, *p* = 0.151, Table 4) associated with a higher potassium intake (potassium score = 1, >2,238 mg/1,000 kcal) in our study population. The use of potassium-enriched salt instead of regular salt has been shown to decrease cardiovascular mortality in an elderly population (42). An increase in potassium intake may improve blood pressure control (43) and decrease cardiovascular disease risk (44). In an animal model of non-ischemic heart failure (45), a potassium-supplemented diet alleviated cardiorespiratory dysfunction compared with a normal diet. The possible benefit of potassium-enriched salt in patients with heart failure awaits further studies. Likewise, more research is needed to explore the potential benefits of magnesium (46, 47) and fiber (48) intakes on all-cause mortality in heart failure.

This study has several limitations. First, this was an observational study. Our findings need to be confirmed in interventional studies. Second, information on nutritional intakes was obtained from a 24-h dietary recall interview. There might be some variations between the information from the 24-h recall and long-term dietary patterns. Third, we did not have information on left ventricular ejection fraction and N-terminal pro-brain natriuretic peptide, as well as medical treatment for some chronic diseases, such as hypertension and heart failure. This should be taken into account when interpreting our results. Last, the median DASH score was only two in the study population. This indicates that the degree of concordance with the DASH diet was low, which might help explain the null findings in this study. More evidence is required for specific recommendations of dietary components to improve outcomes in patients with heart failure. In this context, our results may have implications for future investigations.

In conclusion, a higher DASH score (>2 vs. ≤ 2) was not associated with all-cause mortality in patients with heart failure. Notably, a decrease in sodium intake (sodium score = 1) was not associated with a significant difference in the risk of mortality. More studies are needed to elucidate the effect of the DASH diet on outcomes in patients with a history of heart failure.

## Data availability statement

Publicly available datasets were analyzed in this study. This data can be found here: https://www.cdc.gov/nchs/nhanes/index.htm.

## Ethics statement

The studies involving human participants were reviewed and approved by the Institutional Review Board of Taichung Veterans General Hospital, Taichung, Taiwan. The patients/participants provided their written informed consent to participate in this study.

## Author contributions

T-YC and J-SW designed and conducted the research and wrote the first draft of the manuscript. W-JL and C-LL analyzed data and revised the manuscript critically for important intellectual content. All authors approved the final draft of the manuscript.

## Funding

This work was supported by Taichung Veterans General Hospital, Taichung, Taiwan (Grant Numbers: TCVGH-1083505C, 2019; TCVGH-1093504C, 2020; TCVGH-1103502C; TCVGH-1103504C; and TCVGH-1107305D, 2021).

## Conflict of interest

The authors declare that the research was conducted in the absence of any commercial or financial relationships that could be construed as a potential conflict of interest.

## Publisher's note

All claims expressed in this article are solely those of the authors and do not necessarily represent those of their affiliated organizations, or those of the publisher, the editors and the reviewers. Any product that may be evaluated in this article, or claim that may be made by its manufacturer, is not guaranteed or endorsed by the publisher.
